# Effect of telmisartan and vitamin E on liver histopathology with non‐alcoholic steatohepatitis: A randomized, open‐label, noninferiority trial

**DOI:** 10.1002/jgh3.12315

**Published:** 2020-03-02

**Authors:** Shahinul Alam, Mushfiqul Abrar, Saiful Islam, Mohammad Kamal, Mohammad J Hasan, Md. Abdullah S Khan, Nooruddin Ahmad

**Affiliations:** ^1^ Department of Hepatology Bangabandhu Sheikh Mujib Medical University Dhaka Bangladesh; ^2^ Department of Pathology Bangabandhu Sheikh Mujib Medical University Dhaka Bangladesh; ^3^ Department of Medicine Pi Research Consultancy Center Dhaka Bangladesh; ^4^ Department of Medicine Shaheed Sayed Nazrul Islam Medical College Kishoreganj Bangladesh

**Keywords:** non‐alcoholic steatohepatitis, telmisartan, vitamin E

## Abstract

**Background and Aim:**

To compare the effect of telmisartan and vitamin E on liver histopathology of non‐alcoholic steatohepatitis (NASH) patients.

**Methods:**

This noninferiority clinical trial was conducted for 1 year. Fatty liver patients with non‐alcoholic fatty liver disease (NAFLD) activity score (NAS) ≥ 5 (in liver biopsy) were selected. All methods were in accordance with the Declaration of Helsinki. Patients who received telmisartan and vitamin E were denoted as Group‐T and Group‐E, respectively. Forty patients >18 years old were assigned and divided into two groups (20 in each group). Histological improvements were primary outcome measures.

**Results:**

Significant improvement in NAS score was noted in both groups (Group E [GE]: 6 ± 0.8 to 4.36 ± 1.4; *P* = 0.00 and Group T [GT]: 5.6 ± 0.7to 4.9 ± 1.2; *P* = 0.03). Fibrosis score improved from 1.6 ± 0.5 to 1.5 ± 0.5 in GE and from 1.7 ± 0.9 to 1.5 ± 0.7 in GT (*P* = 0.67 and 0.42, respectively). Steatosis improved in GE from 2.07 ± 0.6 to 1.14 ± 0.66 (*P* = 0.00) and in GT from 1.94 ± 0.57 to 1.56 ± 0.8 (*P* = 0.05). Lobular inflammation improved from 2.0 ± 0.4 to 1.6 ± 0.5 in GE (*P* = 0.02) and from 1.9 ± 0.3 to 1.8 ± 0.4 in GT (*P* = 0.58). Ballooning score in GE decreased from 1.9 ± 0.3 to 1.7 ± 0.5 (*P* = 0.03), and in GT, it reduced from 1.9 ± 0.1 to 1.5 ± 0.5 (*P* = 0.19). NAS improvement was similar in GE (1.6 ± 1.2) and GT (0.6 ± 1.1; *P* = 0.07) when controlled for weight reduction.

**Conclusion:**

Telmisartan was similar to vitamin E in improving the histology of NASH patients.

## Graphical abstract

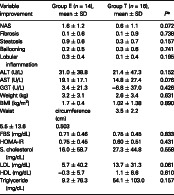

Vitamin E was thought to be effective for non‐alcoholic steatohepatitis (NASH), but this study proved that telmisartan is also comparable to vitamin E for NASH with few admitting limitations.

## Introduction

Non‐alcoholic fatty liver disease (NAFLD), a hepatic manifestation of metabolic syndrome, affects 20–30% of the general population.^1^, ^2^, ^3^ An ample amount of literature has highlighted NAFLD as a global epidemic^4^ with diverse prevalence rates, including 20–30%,^5^ 5–24%,^1^ and 16–32%^4^ in Europe, China, and India, respectively. Recent statistics showed that the prevalence of NAFLD in Bangladesh is as high as around 34.34%, which is much higher than Hepatitis B (4.9%) and Hepatitis C (0.2%).^6^

In adults, NAFLD is typically classified into two categories: non‐alcoholic fatty liver (NAFL) and non‐alcoholic steatohepatitis (NASH).^7^, ^8^ NAFL is generally considered a benign condition, but it can progress to more advance liver disease, such as cirrhosis and hepatocellular carcinoma.^8^, ^9^, ^10^ However, a major impediment of the prevention of the disease was less understanding of underlying pathogenesis.^11^, ^12^, ^13^ Therefore, currently no effective treatment is available for NASH.^14^ Most hepatologists attempt to manage NASH through lifestyle changes, as well as standard therapeutic interventions to control concomitant disease, for example, hyperlipidemia, hypertension, and type 2 diabetes mellitus.^14^, ^15^

As oxidative stress has been implicated in the pathogenesis of NAFLD, the role of antioxidants such as vitamin E, which is known to react with reactive oxygen species (ROS), blocking the propagation of free radical reactions in a wide range of oxidative stress situations, has been consequently tested on several occasions.^16^ These studies demonstrated improvement in biochemical profiles, with a decline in or normalization of liver enzymes. Furthermore, histological assessment showed favorable outcomes in lobular inflammation and hepatic steatosis following treatment with vitamin E. Therefore, vitamin E has been recommended in current American guidelines for treating NASH.^17^ However, the recommendation is limited to only nondiabetic adults with biopsy‐proven NASH.^17^, ^18^ As a result, finding newer treatment options for all NASH patients is a concern of scientists.

Telmisartan, a potent antagonist of the angiotensin II type‐1 (AT1) receptor, usually indicated for essential hypertension, has recently been proven to act against oxidative stress, insulin resistance, and hepatic fibrogenesis.^19^, ^20^ Human studies with angiotensin receptor blocker (ARB) in NASH are few but promising. In Bangladesh, Alam *et al*.^21^ investigated the effect of telmisartan on histological activity and fibrosis in NASH patients and observed that telmisartan significantly improves both NAFLD activity score (NAS) and fibrosis score. Considering the study's limitations, it planned to evaluate the comparative superiority of drug, which may pave the way for the management of NASH patients in the world.

## Methods

### *Study design, selection process, and data collection procedure*

This randomized, open‐label, noninferiority clinical trial was conducted in the Department of Hepatology, Bangabandhu Sheikh Mujib Medical University (BSMMU), for the first time from July 2016 to June 2017. Before commencement of the study, it was ethically approved by Institutional Review Board (IRB) of BSMMU. The clinical trial was registered on the Sri Lanka Clinical Trials Registry (Registration no. SLCTR/2016/013; date: 8 June 2016). Patients with ultrasonogramme (USG)‐proven NAFLD admitted to the Department of Hepatology were screened and approached for index biopsy of the liver. Evidence of steatosis, lobular inflammation, and hepatocellular ballooning in biopsy were considered essential components for the diagnosis of NASH. Patients aged >18 years with NAS ≥5 in liver histology were considered for inclusion in the study. On the other hand, patients with a history of alcohol intake >20 g/day; a history of consumption of drugs that can cause fatty liver, that is, tamoxifen, valproic acid, amiodarone, and methotrexate; or a history of drugs that have shown benefit in previous NASH pilot studies, that is, metformin, thiazolidinediones, and fibrates, were considered for exclusion. Moreover, patients with chronic liver disease (CLD) with known etiology (due to hepatitis B virus (HBV), hepatitis C virus (HCV), Wilson's disease, drug‐induced liver injury etc.); pregnancy; comorbid conditions such as chronic obstructive pulmonary disease (COPD), chronic kidney disease (CKD), and congestive cardiac failure (CCF); and history of recent myocardial infarction (MI) were also excluded from the study. Randomization was carried out by lottery method. For the convenience of the study, total study population was divided into two groups (1:1), named: Group T and Group E. Group T patients received 40 mg of telmisartan once daily, and Group E received 800 IU vitamin E for a similar duration. Besides the standard treatment, lifestyle modification was advised for both groups of patients. Patient were advised to avoid saturated fat, excessive sugar‐containing diet, soft drinks, fast food, and refined carbohydrates and were also encouraged to perform moderate exercise (walking 30 min/day). Diabetic patients were treated with lifestyle modification and, if required, with oral sulphonylureas—gliclazide, glimeperide—or with insulin. Dyslipidemia was managed with statin, and for hypertension, antihypertensive drugs, except angiotensin converting enzyme (ACE) inhibitor, ARB, and calcium channel blocker (Diltiazem), were used.

**Figure 1:**
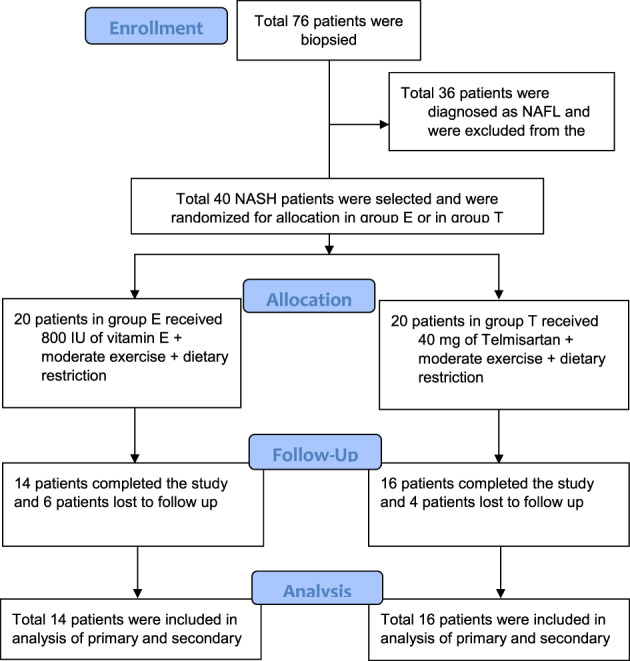
Flow chart of patient selection. NAFL, non‐alcoholic fatty liver; NASH, non‐alcoholic steatohepatitis.

Within the study period, 76 NAFLD patients underwent liver biopsy, and 40 were diagnosed with NASH with an NAS score ≥5. Then, they were randomly allocated to the two previously mentioned groups, with 20 patients in each group. Each patient was advised to attend monthly follow up for 3 months and then follow up every 3 months for the next 9 months. At the end of 1‐year follow up, the total number of attritions was 10 (4 patients in Group T and 6 patients in Group E) due to unwillingness to undergo liver biopsy and lack of interest (for details, see Figure 1).

During enrollment of the study, baseline information was collected and recorded in a separate case record form. All necessary investigations were performed and recorded. In the entire period of observation, an alcohol consumption questionnaire was administered at each visit, and study compliance was strictly monitored. Fasting blood sugar (FBS), 2 h after breakfast (2HABF) and fasting lipid profile for diabetic and dyslipidemia patients were assessed according to need. At the end of 1 year, liver biopsy was repeated in both groups. The primary parameters compared between the first and last visits were vital parameters, blood profile and NAS (including its components such as steatosis, ballooning, and lobular inflammation), and fibrosis score.

Informed consent was obtained from all patients during enrollment of the study and before liver biopsy. Ethical measures were confirmed to be in concordance with the Helsinki declaration.

### *Statistical analysis*

Quantitative data were presented as mean ± SD, and qualitative data were presented in percentage. To determine associations between the categorical variables, chi‐square test was used, and in case of continuous variables, the paired *t* test was used. Comparison between the two groups were performed using an unpaired *t* test. Analysis of covariance (ANCOVA) was used to control for the effect of weight loss on changes in NAS and fibrosis score. The study was conducted with 95% confidence level at 5% acceptable error level, and *P* value <0.05 was considered statistically significant. To account for the decreased power associated with small sample size, we calculated the between‐subject effect size (Cohen's *d*) for the improvement parameter of NAS. A large effect size of >0.8 was found, making our statistical inference relevant. Data analysis was performed using SPSS 23 (SPSS Inc, Chicago, USA).

This study was reviewed and approved by the IRB. BSMMU/2016/3054.

## Results

### *Baseline characteristics of patients*

Forty NASH patients were selected initially. Six patients of Group E were lost to follow up due to lack of interest, and four patients of Group T did not agree to second biopsy (Fig. 1). Thirty NASH patients (14 patients in of Group E and 16 patients in Group T) were considered for final analysis.

The distribution of patients between groups was statistically similar in relation to demographic variables, as well as anthropometric measures, proportion of comorbidity, liver function tests, lipid profile, and glycemic index at baseline. Both groups of patients had an average body mass index (BMI) within the obese range (according to Asian criteria). Baseline liver histology was also statistically similar between groups. Mean NAS was 5.93 ± 0.73 in Group E and 5.63 ± 0.72 in Group T (*P* = 0.262). Mean fibrosis score was 1.57 ± 0.51 in Group E and 1.69 ± 0.70 in Group T (*P* = 0.615) (Table 1).

**Table 1:** Baseline characteristics of the vitamin E and telmisartan groups

Variable	Group E (*n* = 14), mean ± SD	Group T (*n* = 16), mean ± SD	*P*
Age (year)	39.5 ± 9.0	36.6 ± 7.8	0.348
Gender (male: female)	5:9	5:11	1.00
Diabetes (present/absent)	6/8	4/12	0.442
Hypertension (present/absent)	6/8	4/12	0.442
BMI (kg/m^2^)	28.8 ± 3.5	28.9 ± 5.4	0.926
Waist circumference (cm)	98.3 ± 8.0	99.7 ± 17.4	0.532
Steatosis	2.07 ± 0.62	1.94 ± 0.57	0.543
Ballooning	1.86 ± 0.36	1.88 ± 0.34	0.891
Lobular inflammation	2.00 ± 0.39	1.88 ± 0.34	0.359
NAS	5.93 ± 0.73	5.63 ± 0.72	0.262
Fibrosis	1.57 ± 0.51	1.69 ± 0.70	0.615
ALT (U/L)	58.21 ± 39.2	60.7 ± 37.2	0.861
AST (U/L)	42.0 ± 16.6	48.5 ± 28.3	0.459
GGT (U/L)	45.6 ± 22.1	41.8 ± 24.1	0.656
FBS (mmol/L)	6.1 ± 1.6	6.3 ± 2.9	0.822
Blood sugar 2 h after breakfast (mmol/L)	9.9 ± 3.7	10.2 ± 4.7	0.870
Insulin resistance index (HOMA‐IR)	2.4 ± 1.5	2.5 ± 1.6	0.916
Serum cholesterol (mg/dL)	202.5 ± 33.7	213.7 ± 61.5	0.426
HDL (mg/dL)	37.0 ± 5.7	39.1 ± 9.8	0.472
LDL (mg/dL)	120.1 ± 28.7	114.4 ± 33.1	0.539
Triglyceride (mg/dL)	212.2 ± 87	202.9 ± 68.5	0.497

ALT, alanine transaminase; AST, aspartate aminotransferase; BMI, body mass index; FBS, fasting blood sugar; GGT, gamma‐glutamyltrasferase; HDL, high‐density lipoprotein; HOMA‐IR, homeostasis model assessment insulin resistance; LDL, low‐density lipoprotein; NAS, NAFLD activity score.

Table 2 enlists the changes in anthropometry, biochemistry, and liver histology after 1 year of treatment in Groups E and T. Table 3 compares the dynamic characteristic improvement between those treatment groups.

**Table 2:** Changes in anthropometry, biochemistry, and histology after 12 months

	Group E (*n* = 14), mean ± SD			Group T (*n* = 16), mean ± SD		
Variable	Baseline	After 12 months	*P*	Baseline	After 12 months	*P*
Anthropometric changes						
Weight (kg)	71.2 ± 13.0	68.0 ± 13.2	0.002**	68.5 ± 13.1	62.2 ± 8.5	0.008**
BMI (kg/m^2^)	28.8 ± 3.55	27.05 ± 3.9	0.000**	28.9 ± 5.4	27.9 ± 5.9	0.010*
WC (cm)	98.36 ± 7.99	94.79 ± 8.51	0.000**	96.3 ± 9.49	94.19 ± 9.41	0.003**
Liver histology						
Steatosis	2.1 ± 0.6	1.1 ± 0.6	0.000**	1.9 ± 0.5	1.6 ± 0.8	0.054
Ballooning	1.9 ± 0.3	1.5 ± 0.5	0.055	1.9 ± 0.3	1.5 ± 0.5	0.029*
Lobular inflammation	2.0 ± 0.4	1.6 ± 0.5	0.019*	1.9 ± 0.3	1.8 ± 0.4	0.580
NAS	5.9 ± 0.8	4.3 ± 1.4	0.000**	5.6 ± 0.7	4.9 ± 1.2	0.029*
Fibrosis	1.6 ± 0.5	1.5 ± 0.5	0.671	1.7 ± 0.9	1.5 ± 0.7	0.423
Liver biochemistry						
ALT (U/L)	58.2 ± 39.2	27.1 ± 14.4	0.010*	60.7 ± 37.2	39.2 ± 34.1	0.090
AST (U/L)	42 ± 16.6	22.8 ± 7.07	0.001**	48.5 ± 28.3	33.6 ± 24.5	0.046*
GGT (U/L)	43.3 ± 20.8	39.8 ± 26.7	0.606	41.8 ± 24.1	48.6 ± 58.0	0.483
Blood biochemistry						
FBS (mmol/L)	6.1 ± 1.6	5.5 ± 1.5	0.047*	6.3 ± 3.0	5.3 ± 1.1	0.285
Insulin resistance index (HOMA‐IR)	2.4 ± 1.5	1.9 ± 0.5	0.201	2.5 ± 1.7	1.7 ± 0.8	0.097
S. cholesterol (mg/dL)	202.5 ± 33.7	170.0 ± 34.8	0.325	213.7 ± 41.4	186.4 ± 39.9	0.028*
LDL (mg/dL)	120.1 ± 28.7	114.4 ± 33.1	0.619	130.9 ± 34.7	117.1 ± 35	0.125
HDL (mg/dL)	37.0 ± 5.7	36.6 ± 7.8	0.819	39.1 ± 9.8	38.0 ± 7.6	0.483
Triglyceride (mg/dL)	212.2 ± 87	202.9 ± 68.5	0.657	241.7 ± 138.1	187.6 ± 110.2	0.053

For paired samples *t*‐test, *P < 0.05, **P < 0.01 for within‐group comparisons.

ALT, alanine transaminase; AST, aspartate aminotransferase; BMI, body mass index; FBS, fasting blood sugar; GGT, gamma‐glutamyltrasferase; HDL, high‐density lipoprotein; HOMA‐IR, homeostasis model assessment insulin resistance; LDL, low‐density lipoprotein; NAS, NAFLD activity score; WC, waist circumference.

**Table 3:** Dynamic characteristic improvement between vitamin E and telmisartan groups

Variable improvement	Group E (*n* = 14), mean ± SD	Group T (*n* = 16), mean ± SD	*P* *
NAS	1.6 ± 1.2	0.6 ± 1.1	0.072
Fibrosis	0.1 ± 0.6	0.1 ± 0.9	0.738
Steatosis	0.9 ± 0.6	0.3 ± 0.7	0.157
Ballooning	0.2 ± 0.5	0.3 ± 0.6	0.741
Lobular inflammation	0.3 ± 0.4	0.1 ± 0.4	0.195
ALT (U/L)	31.0 ± 38.8	21.4 ± 47.3	0.152
AST (U/L)	19.1 ± 17.1	14.8 ± 27.4	0.076
GGT (U/L)	3.4 ± 21.3	−6.8 ± 37.0	0.426
Weight (kg)	3.2 ± 3.1	2.6 ± 3.4	0.631
BMI (kg/m^2^)	1.7 ± 0.4	1.02 ± 1.38	0.890
Waist circumference (cm)	3.5 ± 2.2	5.5 ± 13.6	0.503
FBS (mg/dL)	0.71 ± 0.46	0.75 ± 0.45	0.833
HOMA‐IR	0.75 ± 0.45	0.60 ± 0.51	0.431
S. cholesterol (mg/dL)	16.0 ± 58.7	27.3 ± 44.8	0.558
LDL (mg/dL)	5.7 ± 40.2	13.7 ± 31.3	0.061
HDL (mg/dL)	−0.3 ± 5.7	1.1 ± 6.6	0.610
Triglyceride (mg/dL)	9.2 ± 76.3	54.1 ± 103.0	0.157

* *P* value for histological improvement was obtained by analysis of covariance (adjusted for improvement in BMI and waist circumference) and by unpaired samples *t*‐test for other dynamic characteristics.

ALT, alanine transaminase; AST, aspartate aminotransferase; BMI, body mass index; FBS, fasting blood sugar; GGT, gamma‐glutamyltrasferase; HDL, high‐density lipoprotein; HOMA‐IR, homeostasis model assessment insulin resistance; LDL, low‐density lipoprotein; NAS, NAFLD activity score.

### *Histological improvement*

NAS improvement was statistically significant in Group E (5.9 ± 0.8 to 4.3 ± 1.4, *P* < 0.001) and Group T (5.6 ± 0.7 to 4.9 ± 1.2, *P* = 0.029). In Group E, histology improved significantly in all components of NAS except ballooning, where the improvement was not statistically significant. In Group T, histologic improvement was noted in all components, but significant improvement was noted only in ballooning. Fibrosis score also improved in both groups. Therefore, Group E patients showed significant improvement in steatosis, lobular inflammation, and overall NAS, and Group T showed significant improvement in ballooning and overall NAS (Table 2). At the end of the study, dynamic improvement in NAS was statistically similar in Groups E and T when statistically adjusted for weight reduction (1.6 ± 1.2 *vs* 0.6 ± 1.1; *P* value = 0.072). Comparison of individual components of the NAS score between the treatment groups shows that improvement in steatosis was significantly higher in Group E than Group T (*P* = 0.033). However, in ballooning and lobular inflammation scores, mean improvement did not differ significantly between Groups E and T (*P* = 0.654 and *P* = 0.432, respectively). In Group E, mean fibrosis score improvement was 0.1 ± 0.6, whereas in Group T, it was 0.1 ± 0.9. The difference in fibrosis score improvement between Groups E and T was not statistically significant (*P* = 0.580) (Table 3).

### *Anthropometric changes*

Both groups showed statistically significant improvement in weight and BMI after the end of treatment (Table 2). Mean weight improvement in Groups E and T were 3.2 ± 3.1 and 2.6 ± 3.4, respectively (*P* = 0.631). Mean improvement in BMI was 1.7 ± 0.4 in Group E and 1.02 ± 1.38 in Group T (*P* = 0.89) (Table 3). Therefore, both groups achieved similar improvement in weight and BMI of patients.

### *Changes in liver biochemistry*

Regarding changes in liver biochemistry, Group E showed significant reduction in alanine transaminase (ALT) and aspartate aminotransferase (AST), but reduction in gamma‐glutamyltrasferase (GGT) level was not statistically significant. Group T also showed reduction in ALT and AST, with reduction being significant in the latter. While GGT level increased in Group T (Table 2), mean changes in ALT, AST, and GGT did not differ significantly between Groups E and T (Table 3).

### *Changes in glycemic profile and serum lipid profile*

FBS reduced statistically significantly from baseline in both groups after treatment. Homeostasis model assessment insulin resistance (HOMA‐IR) showed nonsignificant improvement in both groups. All components of the serum lipid profile showed improvement with treatment in both groups, with serum cholesterol level showing significant decrease in patients receiving telmisartan (Table 2).

### *Comparison between histological responders and nonresponders*

NAS ≥2 improvement without worsening of the fibrosis score was considered a histological responder. Accordingly, 7 patients (23.3%) were responders, and 23 patients (76.7%) were nonresponders. Among responders, five patients (71.4%) were in Group E, and two patients (28.6%) were in Group T. The difference in responses between Groups E and T was not statistically significant (*P* = 0.204).

Table 4 shows the comparison of baseline factors between responders and nonresponders. Mean age and male–female ratio were similar in both responder and nonresponder groups. In addition, baseline BMI, waist circumference, and FBS did not differ in two groups, except 2HABF. Mean 2HABF was 7.1 ± 1.9 mmol/L and 10.8 ± 4.2 mmol/L in responders and nonresponders, respectively (*P* = 0.045). Baseline lipid profile was similar between responders and nonresponders, except high‐density lipoprotein (HDL). Mean HDL differed significantly between responders and nonresponders (30.5 ± 3.6 and 40.1 ± 7.8 mg/dl, respectively; *P* = 0.007). Baseline liver function tests were also statistically similar among responders and nonresponders. Mean NAS score at baseline was 6 ± 0.8 and 5.7 ± 0.7 in responders and nonresponders, respectively. Mean fibrosis score at baseline was 1.5 ± 0.5 in responders and 1.6 ± 0.6 in nonresponders.

**Table 4:** Comparison of baseline factors between responders and nonresponders

Baseline factors	Responders (*n* = 7), mean ± SD	Nonresponders (*n* = 23), mean ± SD	*P*
Category of patients (Group E/Group T)	5/2 (71.4%/28.6%)	9/14 (39.1%/60.9%)	0.204
Age (in years)	36.0 ± 11.5	38.5 ± 7.6	0.525
Gender (male/female)	4/3 (57.1%/42.9%)	6/17 (26.1%/73.9%)	0.181
Obesity (present/absent)	6/1 (85.7%/14.3%)	19/4 (82.6%/17.4%)	1.00
Waist circumference increased (yes/no)	6/1 (85.7%/14.3%)	22/1 (95.7%/4.3%)	0.418
Diabetes mellitus (yes/no)	0/7 (0%/100%)	10/13 (43.5%/56.5%)	0.064
Hypertension (yes/no)	1/6 (14.3%/85.7%)	9/14 (39.1%/60.9%)	0.372
BMI (kg/m^2^)	26.6 ± 2.1	29.4 ± 4.9	0.182
Waist circumference (cm)	94.0 ± 2.9	98.0 ± 9.5	0.314
FBS (mmol/L)	5.3 ± 0.7	6.4 ± 2.6	0.305
2HABF (mmol/L)	7.1 ± 1.95	10.89 ± 4.27	0.045^a^
Cholesterol (mg/dL)	192.3 ± 29.7	212.5 ± 39.1	0.249
LDL (mg/dL)	115.1 ± 31.65	126.7 ± 32.6	0.442
HDL (mg/dL)	30.5 ± 3.6	40.1 ± 7.8	0.007^b^
TG (mg/dL)	204.5 ± 82.1	233.8 ± 123.4	0.589
ALT (U/L)	78.6 ± 53.1	54.7 ± 32.2	0.167
AST (U/L)	49.1 ± 17.4	44.5 ± 24.9	0.673
GGT (U/L)	42.1 ± 23.2	43.9 ± 23.3	0.868
HOMA‐IR	1.8 ± 0.8	2.6 ± 1.6	0.302
NAS score	6 ± 0.8	5.7 ± 0.7	0.482
Fibrosis score	1.5 ± 0.5	1.6 ± 0.6	0.562

For unpaired samples *t*‐test, *P < 0.05, **P < 0.01.

2HABF, 2 h after breakfast; ALT, alanine transaminase; AST, aspartate aminotransferase; BMI, body mass index; FBS, fasting blood sugar; GGT, gamma‐glutamyltrasferase; HDL, high‐density lipoprotein; HOMA‐IR, homeostasis model assessment insulin resistance; LDL, low‐density lipoprotein; NAS, NAFLD activity score; TG, triglyceride.

Table 5 shows that the average improvement in FBS and 2HABF between the two groups was not statistically significant. The mean improvement in HOMA‐IR, fasting lipid profile, and liver function tests was not significantly different either. Mean BMI improvement was 1.9 ± 0.5 kg/m^2^ and 1.2 ± 1.3 kg/m^2^ (*P* = 0.154), and mean waist circumference improvement was 4.2 ± 2.6 cm and 2.3 ± 2.1 cm (*P* = 0.060) in responders and nonresponders, respectively. Weight loss of ≥5% occurred in 13 (43.3%) of 30 patients. Five patients (71.1%) among histological responders and eight patients (34.8%) among histological nonresponders had weight reduction of ≥5%. The difference was not statistically significant (*P* = 0.087). On the other hand, ≥7% weight loss was achieved in eight patients (26.7%). Among them, three patients were histological responders (42.9%), and five were histological nonresponders (21.7%). Thus, weight improvement of ≥7% was similar across groups (*P* = 0.300). None of the patients in this study achieved 10% weight loss.

**Table 5:** Comparison of dynamic factor improvement between responders and nonresponders

Dynamic factors improvement	Responders (*n* = 7), mean ± SD	Nonresponders (*n* = 23), mean ± SD	*P*
Weight reduction ≥5% (yes/no)	5/2	8/15	0.087
Weight reduction ≥7% (yes/no)	3/3	5/19	0.300
Waist circumference (cm)	4.2 ± 2.6	2.3 ± 2.1	0.060
BMI (kg/m^2^)	1.9 ± 0.5	1.2 ± 1.3	0.154
FBS (mmol/L)	0.2 ± 0.9	0.9 ± 2.9	0.511
2HABF (mmol/L)	0.6 ± 1.7	1.9 ± 4.6	0.481
Total cholesterol (mg/dL)	12.4 ± 55.2	25.0 ± 50.8	0.579
LDL (mg/dL)	8.4 ± 37.1	10.3 ± 35.8	0.905
HDL (mg/dL)	1.2 ± 3.8	−1.4 ± 6.5	0.311
TG (mg/dL)	2.1 ± 84.9	42.6 ± 94.8	0.321
ALT (U/L)	48.8 ± 48.9	18.9 ± 39.7	0.110
AST (U/L)	25.7 ± 16.8	14.1 ± 24.1	0.250
GGT (U/L)	6.2 ± 8.03	−4.2 ± 34.6	0.561
HOMA‐IR	0.08 ± 1.2	0.84 ± 1.6	0.577

2HABF, 2 h after breakfast; ALT, alanine transaminase; AST, aspartate aminotransferase; BMI, body mass index; FBS, fasting blood sugar; GGT, gamma‐glutamyltrasferase; HDL, high‐density lipoprotein; HOMA‐IR, homeostasis model assessment insulin resistance; LDL, low‐density lipoprotein; TG, triglyceride.

## Discussion

The present open‐label, randomized, noninferiority clinical trial investigated the effects of 1 year of treatment with telmisartan and vitamin E on liver histology in NASH patients. To our knowledge, this study is the first of its kind among biopsy‐proven NASH patients.

The most significant changes in hepatic histology induced by vitamin E in this study were reflected by the attenuation of steatosis, lobular inflammation, and NAS. These findings are consistent with Sanyal *et al*.,^22^ who observed in the PIVENS trial that vitamin E reduces steatosis and lobular inflammation. In addition, they reported significant improvement in hepatocyte ballooning, which was not significant in the current study. Improvement of fibrosis score was not significant in our study and in the PIVENS trial. Lavine *et al*.,^23^ in their multicenter, randomized clinical trial on pediatric NAFLD patients, found no significant changes in fibrosis, degree of inflammation, or steatosis independent of the clinical center. They observed improvements in resolution of NAS and hepatocellular ballooning. However, one fact is common in every study: vitamin E does not reduce fibrosis score. Therefore, we can infer that vitamin E is beneficial in improving NAS but not effective for fibrosis improvement.

With regard to telmisartan, this study demonstrated statistically significant NAS improvement (*P* = 0.029). This finding was consistent with previous studies, such as the one by Georgescu and Ionescu^24^ and Alam *et al*.^21^; both these studies showed a reduction in overall NAS at the end of therapy with telmisartan. However, in contrast to the study by Alam *et al*.,^21^ fibrosis score did not show significant improvement in our study. Alam *et al*. observed that telmisartan improves all components of NAS, as well as fibrosis score.^21^ However, in this study, a higher number of Group T patients showed improvement in fibrosis score >1 in comparison to Group E patients. In the study by Alam *et al*.,^21^ histopathological reports were carried out by different pathologists, but in the current study, all the histopathological reports were carried out by a single expert pathologist to curtail observer variation. This could be the reason behind the inconsistencies between these two studies. Moreover, telmisartan was found to reduce hepatic fibrosis significantly in rats.^25^ Therefore, the effect of telmisartan on hepatic fibrosis in humans has to be consolidated in a further larger‐scale trial.

In Group E, the improvement in ALT and AST at the end of 12 months from baseline was statistically significant (*P* = 0.010 and *P* = 0.001, respectively). These findings are consistent with several previous studies.^23^, ^26^ Sanyal and coworkers, in the PIVENS study,^23^ found that there was an early and highly significant decrease in mean ALT and AST levels among subjects receiving vitamin E. Hasegawa *et al*.,^26^ in 2001, in a pilot study, observed a significant improvement in serum ALT, AST, alkaline phosphatase (ALP), and GGT levels after 1 year of a‐tocopherol treatment at a dose of 300 mg/day. Sanyal and coworkers^23^ also observed that serum concentrations of ALP and γ‐glutamyl transpeptidase improved with use of vitamin E. In the current study, in Group E, although both ALP and GGT reduced at the end of the study, these were not statistically significant.

In the telmisartan group, baseline ALT and AST improved after 12 months of treatment, with the latter reaching statistical significance. Similarly, Alam *et al*.^21^ noted an improvement in the ALT level of telmisartan‐treated patients, although the improvement was not significantly different than that of control arm. Several studies have shown that treatment with telmisartan resulted in a significant improvement in glucose metabolism in insulin‐resistant subjects.^27^, ^28^ However, in our study, blood glucose level and HOMA‐IR did not show significant improvement. Georgescu, Ionescu, and Niculescu, in 2009,^24^ in a randomized clinical trial in biopsy‐proven NASH comparing the effect of telmisartan and valsartan, demonstrated a significant effect on the lipid profile by telmisartan, whereas valsartan seemed to lack this property. Our study also demonstrated significant improvement of serum cholesterol level compared to baseline (*P* = 0.028) in the telmisartan group. Other parameters of lipid profile did not improve significantly in either Group E or Group T.

In our study, on direct head‐to‐head comparison of the improvement of dynamic factors between vitamin E and telmisartan, we found that telmisartan was similar to vitamin E with regard to improvement of overall NAS and fibrosis score (*P* value 0.072 and 0.738, respectively). As lifestyle intervention was adhered to by patients in both groups, and improvement in BMI and weight circumference occurred in both groups, these factors were weighted in the analysis of improvement in the histological activity between these groups.

The improvement in NAS in both groups could be attributed to weight reduction in both groups. In order to adjust the effect of weight reduction, we used ANCOVA for statistical analysis of histological improvement and found that mean improvement in NAS was statistically similar across groups. Therefore, vitamin E and telmisartan both showed similar histological improvement in NASH.

The study limitations were the small sample size, the high number of dropouts, and single center‐based inclusion of subjects. It is very difficult to predict treatment response confidently with a small sample size. The present study also suffered from a lack of multicenter, multiethnic categories of patients.

In conclusion, vitamin E and telmisartan improve NAS but not fibrosis score. Telmisartan was similar to vitamin E in improving the overall histology of NASH patients. We recommend conducting large multicenter, double‐blinded randomized controlled trials to consolidate the findings of this study.
